# Strain–Phonon Cooperation as a Necessary Ingredient to Understand the Jahn–Teller Effect in Solids

**DOI:** 10.1021/acs.jpclett.4c01256

**Published:** 2024-06-13

**Authors:** Toraya Fernández-Ruiz, Inés Sánchez-Movellán, Juan María García-Lastra, Miguel Moreno, José Antonio Aramburu, Pablo García-Fernández

**Affiliations:** † Departamento de Ciencias de la Tierra y Física de la Materia Condensada, Universidad de Cantabria, Cantabria Campus Internacional, Avenida de los Castros s/n, 39005 Santander, Spain; ‡ Department of Energy Conversion and Storage, Technical University of Denmark, 2800 Kgs. Lyngby, Denmark

## Abstract

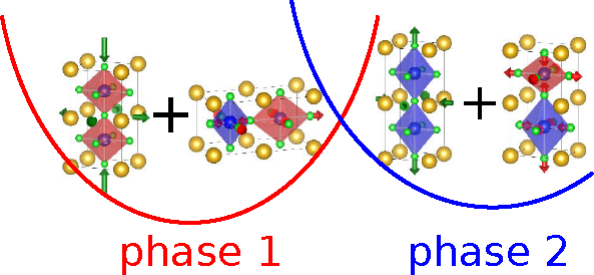

Spatial degeneracy is the cause of the complex electronic, geometrical, and magnetic structures found in a number of materials whose more representative example is KCuF_3_. In the literature the properties of this lattice are usually explained through the Kugel–-Khomskii model, based on superexchange interactions. Here we provide rigorous theoretical and computational arguments against this view proving that structural and magnetic properties essentially arise from electron–vibration (vibronic) interactions. Moreover, based on the work of Öpik and Pryce, we show that the coupling between lattice (homogeneous strain) and motif (phonons) distortions is essential to understand the main stable configurations of the lattice. Using this information, we predict a new low-energy phase in KCuF_3_ that could strongly alter its properties and provide guidance on how to stabilize it through strain engineering.

Spatial degeneracy, the situation where two electronic states have different electron distributions and the same energy, is an important characteristic of quantum systems and strongly influences the properties of materials where it is present.^1,2^ Systems that display electron degeneracy in a high-symmetry, parent geometry have been associated with exotic magnetic structures^3^ and colossal magnetoresistance^4^ and were part of the inspiration behind the discovery of high-temperature cuprate superconductors.^5^ While its description in molecular systems is well understood and fully characterized using group theory and many-body quantum chemistry methods,^1,2,6^ in solids the state of the art is quite different. The main issue is the strongly coupled nature of the electronic, magnetic, and structural degrees of freedom and the determination of which of the possible interactions between them is the key responsible for the observed phenomena in these complex materials. A typical case considers the substitution of Zn^2+^ ions in cubic KZnF_3_ by Cu^2+^, which have similar ionic radii,^7^ to form the parent, cubic phase of KCuF_3_. In this case the crystal contains a lattice of locally degenerate *e*_*g*_ levels, a situation typically solved using orbital ordering approaches, where it is assumed that the local orbitals are free to rotate and the energy of the system is given in terms of interactions between neighboring orbitals, leading to a Heisenberg-like effective Hamiltonian.^3,8−13^ These methods were originally formulated using magnetic interactions,^8,9^ although some of them were later written in terms of electron–vibration coupling,^11,12^ essentially leading to cooperative Jahn–Teller (JT) models.^2,13,14^ Simulations in layered perovskites^15^ have provided quantitative evidence that, although both interactions can be important to reproduce particular properties, electron–vibration is clearly dominant in energetic terms with superexchange playing a small, secondary role. Pavarini et al.^16^ reached similar conclusions for KCuF_3_, although they found a sizable superexchange contribution. However, no fundamental theoretical arguments, on the basic foundations of each of the contributions, have been provided to rely on one or the other. The first goal of this letter is to show that superexchange-based models are supported neither by basic theory, as they conflict with the many-body Bloch theorem, nor by calculation, as their contribution to the stabilization energy is negligible with respect to vibronic coupling. The second goal is associated with the correct characterization of the geometry and electronic structures of these systems. Cooperative JT^2,14^ and orbital ordering models^3,8,9^ provide, for KCuF_3_ and similar systems like KCrF_3_, an antiferro-ordered solution (see Figure 1d). This characteristic pattern first appeared in the treatment of the cooperative JT effect by Kanamori,^14^ who considered two competing situations to solve the problem of KCuF_3_: (i) the antiferrodistortive mode shown in Figure 1d and (ii) the effect of an homogeneous strain that produced the same local JT distortion in all the sites of the lattice (Figure 1a,b). The argument used by Kanamori, echoed by most later authors,^3^ is that the antiferrodistortive mode is the main distortion, as to not to incur in elastic energy penalties associated with strain deformations. In this work, we will reframe the problem of spatial degeneracy in solids using symmetry and first-principles simulations to show that this dichotomy is not correct.

**Figure 1:**
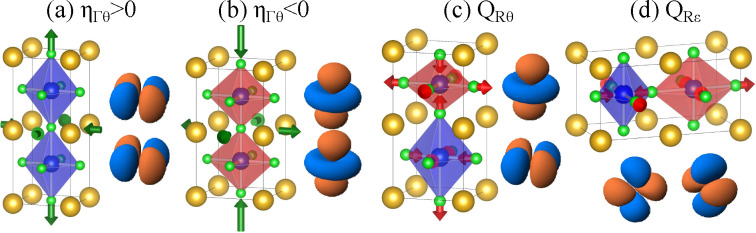
Illustration of the various distortions and possible orbital ordering (unpaired holes) in a perovskite crystal like KCuF_3_. (a) and (b) show the positive (elongation)/negative (compression) tetragonal distortions and orbital ordering associated with homogeneous e_*g*_-strain (η_Γθ_). (c) and (d) illustrate, respectively, the tetragonal (Q_*Rθ*_) and orthorhombic (Q_*Rε*_) R-point antiferrodistortive phonon modes and their associated antiferro-type orbital ordering.

Superexchange-based orbital-ordering, also known as the Kugel–Khomskii model,^3,8,9^ writes the magnetic interactions, based on Anderson’s model,^17^ between the degenerate d-orbitals at the high-symmetry geometry (usually cubic). In cubic lattices, this model allowed to predict^8^ that the ground state of KCuF_3_ was antiferromagnetic A (AF-A) where the holes form a checkerboard (antiferrodistortive) pattern of *x*^2^ – *z*^2^/*y*^2^ – *z*^2^ orbitals (see Figure 1d). From this point of view, the distortion of the lattice is secondary and would simply follow the ordering of the orbitals.^3,9^ However, this kind of solution is contrary to cubic symmetry. According to the many-electron Bloch theorem^18,19^ when applying a translation associated with a cubic lattice vector, , the many-body wave function, , should only change in a phase factor,
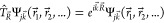
1meaning that in a cubic situation the electron density in all sites is the same and, as a consequence, the orbitals should be aligned in a ferro situation. This indicates that the solution of the Kugel–Khomskii model is symmetry-broken, and it is only valid after the system has been distorted. Fundamentally, this result means that the origin of the distortion in a system with spatial degeneracy can only be the JT or other vibronic effects,^1^ although this statement does not limit the contributions of other phenomena (like superexchange) that may affect the stability of the final low-symmetry configuration. Similar to the Kugel–Khomskii model, DFT simulations (involving LDA+U or hybrid functionals) often lead (see in ref (20)) to symmetry-broken states which hinder reconstructing the energy surface close to the cubic configuration. This finding is in agreement with the results of Varignon and Zunger^21,22^ that prove that DFT solutions are reliable but only at low-symmetry, large-supercell situations. We have checked that all the energy surfaces presented in the present manuscript, and the wave functions associated with them, fulfill all basic symmetry requirements (Bloch theorem) according to the space group of the input geometry.

Imposing Bloch’s theorem to a cubic crystal containing d^9^ ions, like Cu^2+^ in the parent phase of KCuF_3_, we find that there are 2 degenerate energy states per magnetic ordering, where local orbitals^1,2^ are, respectively, χ_–_ or χ_+_,
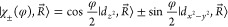
2It is important to note that, given that Bloch’s theorem forces ferrodistortive coupling at the cubic geometry, the angle φ is the same in every lattice site. These two degenerate, many-body wave functions form together a crystal *E*_*g*_ state that, according to the *E*_*g*_ ⊗ *e*_*g*_ JT problem,^1,2^ will couple to *e*_*g*_-symmetry distortions. In a *Pm*3̅*m* perovskite crystal, the only points in the first Brillouin zone that have an associated cubic group (*O*_*h*_) are Γ and *R*, although the periodicity of the distortions will be different at each of them. We can see (Figure 1, check also the detailed analysis in ref (23)) that ferrodistortive coupling is associated with homogeneous e_*g*_-strain distortions in Γ (η_Γθ_, η_Γε_) while antiferrodistortive coupling is associated with e_*g*_-phonon modes in *R* (Q_*Rθ*_, Q_*Rε*_) (see Supporting Information for the specific definition of these distortions). In order to establish how these two distortions interact with each other (to determine whether they compete or cooperate) we look at lowest (elastic) third-order anharmonic terms^24^ that arise as the product of a quadratic R-phonon and a linear Γ strain term, namely,
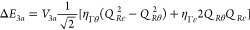
3Although this term may seem somewhat exotic, when the indexes associated with the point of the first Brillouin zone (Γ, *R*) are removed, it reduces to the well-known anharmonic contribution proposed by Öpik and Pryce^25^ to explain why, in most cases, but not always,^26^ octahedral complexes under the JT effect become elongated, a result that was numerically confirmed using first-principles simulations.^24^ The leading term in eq 3 indicates that, depending on the sign of η_Γθ_, a reduction of the energy will occur following either the *Q*_*Rε*_ (when η_Γθ_ < 0, compression of the lattice) or the *Q*_*Rθ*_ (when η_Γθ_ > 0, elongation of the lattice), meaning that lattice (homogeneous strain) and motif (vibration) distortions need to cooperate. A main prediction of this work is that, (i) while the most stable state of pure JT crystals comes from the coupling of η_Γθ_ with *Q*_*Rε*_, leading to a deformation of the octahedral complexes in the xy-plane with alternating long/short metal–ligand distances in the x, y direction (*Q*_*Rε*_, Figure 1d), (ii) there should also exist a second low-energy stable configuration presenting an alternating elongated/compressed complex geometry along the *z*-axis (*Q*_*Rθ*_, Figure 1c). It is of note that Kataoka^27^ also introduced strain–phonon coupling in a cooperative JT effect model, although the symmetry of the resulting terms was different to the present case and did not make, for example, the prediction of the phases proposed here.

After we have established that ferrodistortive strains can cooperate with antiferrodistortive vibration modes, we need to quantify the effect of this cooperation; i.e., we need to check whether ferrodistortive coupling is negligible or is, in fact, significant, requiring a revision of current models. In order to numerically estimate the effect of superexchange, ferro- and antiferrodistortive distortions on the final state of a solid with spatial degeneracy, we have carried out first-principles simulations, involving hybrid DFT functionals and LDA+U using both CRYSTAL and VASP^28,29^ codes (see Supporting Information for details). We chose KCuF_3_ to carry out the calculations since it is one of the most prototypical solid-state systems with orbital degeneracy. At difference with LaMnO_3_, where octahedral tilting plays an important role, in this system there is a consensus that part of the distortion clearly involves the JT effect.^21,22^ We have checked^20^ that the conclusions extracted here are fully generalizable to other JT lattices with very different structures like rocksalt-CuO or bidimensional CuCl_2_. Our main results are presented in Figure 2 and Table 1.

**Figure 2:**
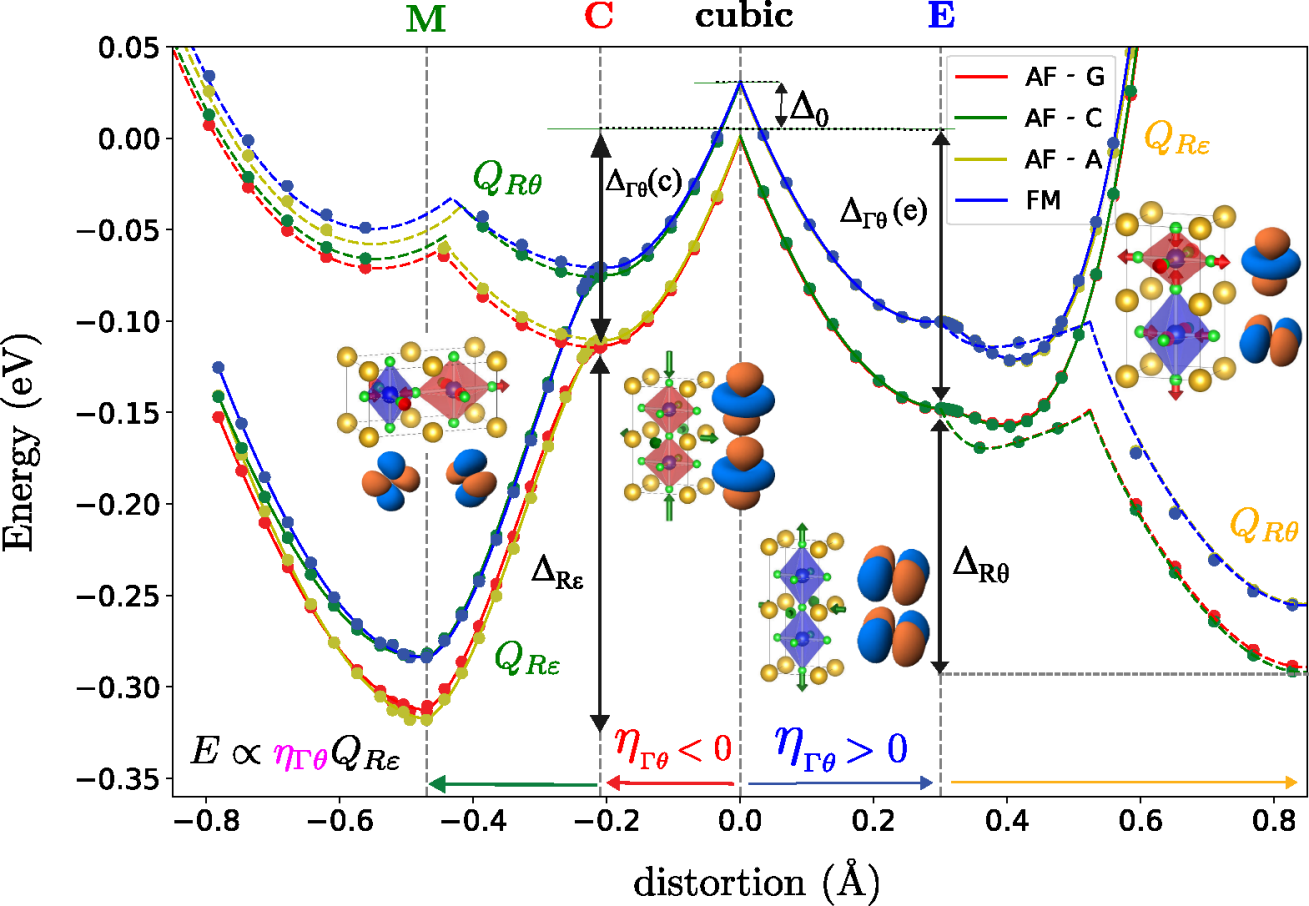
DFT-calculated energy surface of KCuF_3_ in the (η_Γθ_, *Q*_*Rε*_, *Q*_*Rθ*_) space for 4 magnetic states AF-G (red), AF-C (green), AF-A (gold), and FM (blue). The image shows the evolution of the energy from the cubic phase (middle) when the system is compressed (left)/elongated (right) under a homogeneous strain and, successively, the motif is distorted along the two components of the e_*g*_-vibration, *Q*_*Rε*_ (solid lines) or *Q*_*Rθ*_ (dashed lines). The various energies discussed in Table 1 are also shown including initial magnetic energy (Δ_0_), JT energies for elongated/compressed geometries (Δ_Γθ_(*e*)/Δ_Γθ_(*c*)), and compressed to global minimum stabilization energy (Δ_*Rε*_). Orbitals represent unpaired holes.

**Table 1:** DFT-HSE06 Energies (See Figure 2) Involved in the KCuF_3_ Problem Including Initial Magnetic Splitting (Δ_0_), JT Effect Energies Associated with Ferrodistortive Strain Mode η_Γθ_ for Elongated and Compressed Geometries (Δ_Γθ_(*e*)/Δ_Γθ_(*c*)), Stabilization Energy of the *Q*_*Rε*_ Phonon Mode from the Compressed Saddle Point (Δ_*Rε*_(*c*)), Total Distortion Stabilization Energy for the Ground State (Δ_dist_ = Δ_Γθ_(*c*) + Δ_*Rε*_(*c*)), and Total Stabilization Energy Including Magnetic Energy for the Ground State (Δ_Total_ = Δ_dist_ + Δ_0_)^a^

	Δ_0_	Δ_Γθ_(*e*)	Δ_Γθ_(*c*)	Δ_*Rε*_(*c*)	Δ_dist_	Δ_Total_	Δ_Total_^AF-A^	Δ_*Rθ*_(*e*)
FM	31.1	–138.7	–110.3	–224.2	–334.5	–303.4	29.3	–178.9
AF-A	1.2	–137.7	–121.9	–211.9	–333.8	–332.6	0.0	–177.2
AF-C	29.8	–156.1	–113.5	–220.2	–333.7	–303.9	28.7	–152.2
AF-G	0.0	–155.6	–123.2	–205.1	–328.3	–328.3	4.4	–148.8

a On the last column we provide the stabilization energy of the *Q*_*Rθ*_ phonon mode from the elongated saddle point (Δ_*Rθ*_(*e*)) and Δ_Total_^AF-A^ is the final energy with respect to the global AF-A minimum. All energies are given in meV.

First, we studied the effect of the ferrodistortive tetragonal distortion following the η_Γθ_ mode (Figure 1) by optimizing the geometry of KCuF_3_ in the tetragonal *P*4/*mmm* phase. We obtain two saddle points in the energy surface (see Figure 2) that correspond to octahedral elongated (E) and compressed (C) situations along z. In fact, the energy surface contains completely equivalent tetragonal critical points along x and y axes, configuring the (η_Γθ_, η_Γε_) energy surface as the typical warped Mexican hat in JT molecules.^1,24^ Analyzing Mulliken populations, spin spatial distribution, and orbitals at the Γ point, we confirm that the orbitals are, for all magnetic states, ferro-ordered in (η_Γθ_, η_Γε_)-space. As expected from octahedral impurities, for the cases of compressed (elongated) geometries, the unpaired hole orbital has 3*z*^2^ – *r*^2^ (*x*^2^ – *y*^2^) character. The energy surface from the *Pm*3̅m cubic structure to these points can be plotted following the η_Γθ_ strain mode (see Supporting Information for its definition). Our simulations show that the character of the orbitals does not change along this path (φ in eq 2 is fixed), which is the typical behavior^1,2,24^ of JT states in impurities. Thus, we can observe that these two electronic configurations are consistent with the constraints imposed by Bloch’s theorem at the cubic symmetry and the usual E_*g*_ ⊗ *e*_*g*_ JT effect. At the cubic geometry (see Figure 2 and Table 1) the antiferromagnetic G (AF-G) and antiferromagnetic A (AF-A) states are nearly degenerate and below the ferromagnetic (FM) and antiferromagnetic C (AF-C) states by ≈30 meV/formula. The stabilization JT energy, Δ_Γθ_, is somewhat dependent on the (hybrid) functional used, but in all cases we find that it involves a significant energy of ≈110–156 meV/formula which is 4–5 times larger than the separation between magnetic states. Moreover, we find that the JT energy is weaker by ≈10% when the bonds that elongate involve an FM interaction, i.e., the AF-G shows the strongest JT energy both for compression and elongation. After taking into account the distortion associated with the JT effect, η_Γθ_, we allow the system to relax along the antiferrodistortive *Q*_*Rε*_ mode. Starting from the compressed configuration (C in Figure 2) we find a large distortion and relaxation energy, Δ_*Rε*_ of ≈210 meV, that leads to the global minimum (M) with *I*4/*mcm* symmetry. However, if we start from the elongated configuration (E in Figure 2) we find a much smaller distortion and stabilization energy of ≈30 meV along *Q*_*Rε*_. This is a direct proof of the strong coupling between the η_Γθ_ with *Q*_*Rε*_, as depending on the initial η_Γθ_ value the *Q*_*Rε*_ stabilization energy is reduced by more than 80%. Moreover, relaxing only *Q*_*Rε*_ from E does not lead to a true minimum as there exists a strong stress in the η_Γθ_ direction. This can be seen in the leftmost panel in Figure 2 where the energy decreases linearly changing η_Γθ_ for nonzero *Q*_*Rε*_ showing the existence of a term like eq 3 (when η_Γε_ = *Q*_*Rθ*_ = 0). Performing geometry optimization from the elongated configuration with a small *Q*_*Rε*_ distortion leads to a global (compressed) minimum (M). Thus, we can observe that the ferrodistortive mode has a stabilization energy of the same order of the antiferrodistortive one and plays a key role in enhancing the distortion associated with the later.

On the other hand, if, instead of distorting along *Q*_*Rε*_ from E, the *Q*_*Rθ*_ coordinate is followed (dashed lines in Figure 2), we find a very large stabilization energy which is, again, compatible with eq 3. This path leads to a second stable geometry only 25.5 meV above the global minima (see Figure 3) where the complexes distort in an alternating elongated-compressed pattern that is strikingly different from the ground state. Constraining the in-plane lattice parameter to that of the elongated transition state (E) we find that the geometry relaxes mainly along the *Q*_*Rθ*_ distortion showing that this second geometry could be experimentally reached by strain engineering, i.e. growing the crystal on a substrate with a small lattice parameter (*a* ≈ 4.1 Å). It is important to note that this drastic change in the geometry of the system would alter many of the critical properties characteristic of these crystals like polaron motion which, in turn, would affect phenomena like colossal magnetoresistance.

**Figure 3:**
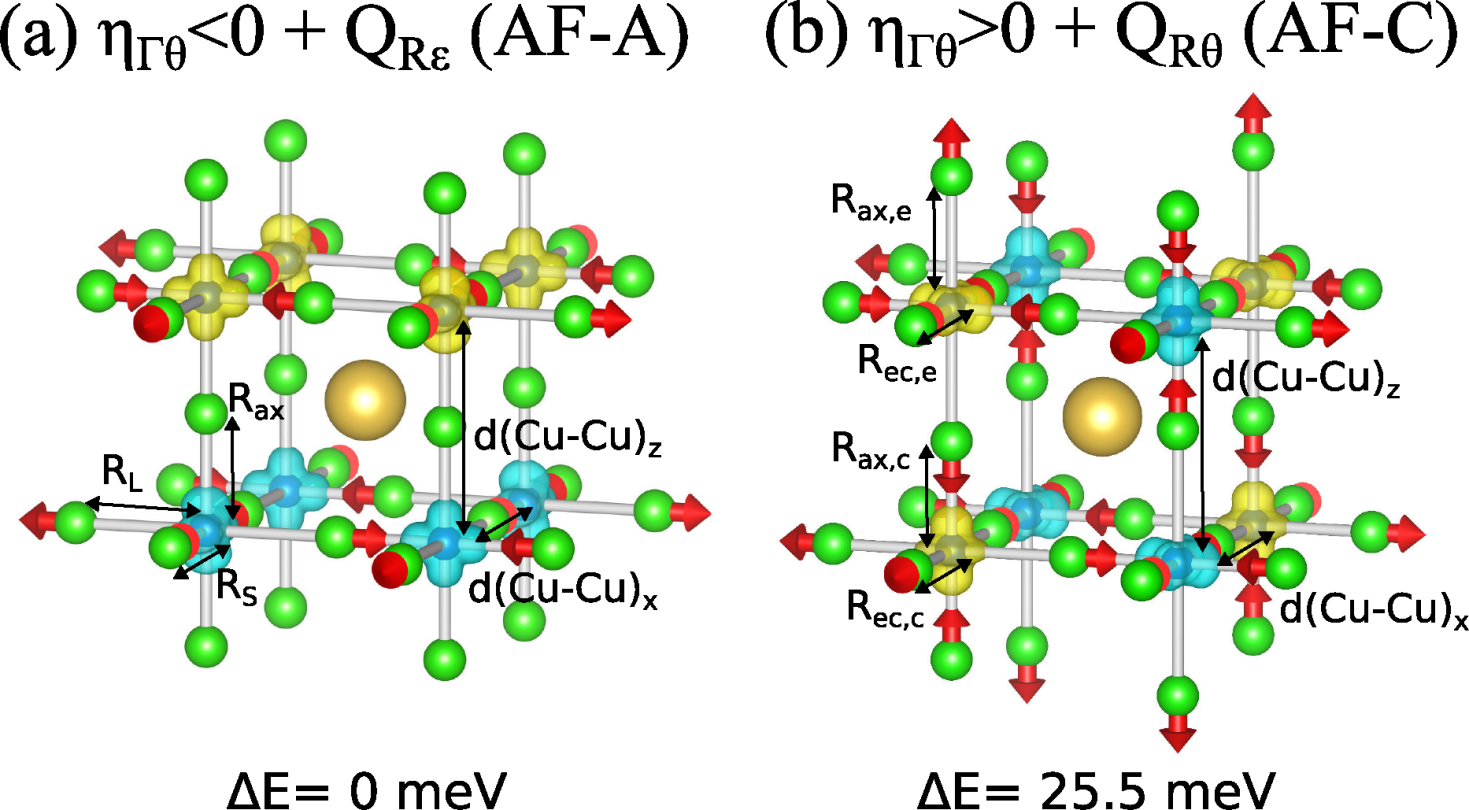
DFT-calculated geometries of the two stable configurations of JT crystals with initial perovskite structure resulting from strain–vibration coupling represented by eq 3. Arrows represent the movement of atoms with respect to the cubic phase while yellow/blue isosurfaces correspond with up/down spin densities. In (a) the local complexes orthorhombic with axial (*R*_*ax*_(z)), long (*R*_*L*_(x/y)), and short (*R*_*S*_(y/x)) metal–ligand distances equal to 1.91, 2.30, and 1.98 Å. In (b) the complexes are tetragonal where the elongated display axial (*R*_*ax*,*e*_) and equatorial (*R*_*ec*,*e*_) distances are equal to 2.30 and 1.94 Å, respectively, while the compressed distances, *R*_*ax*,*c*_ and *R*_*ec*,*c*_, are 1.89 and 2.13 Å.

Studying the evolution of the orbitals along *Q*_*Rε*_, we observe that, at the compressed geometry (C), the orbitals are ferro-ordered, and upon increasing the distortion, the orbitals gain, continuously, some antiferrodistortive character. Importantly, the final local function differs from those of ideal *x*^2^ – *z*^2^/*y*^2^ – *z*^2^ solutions typical of many orbital-ordering models. This behavior is characteristic of the pseudo JT effect where ground and excited state are smoothly mixed by the distortion. This is consistent with the fact that mode *Q*_*Rε*_ belongs to the edge of the first Brillouin zone and has a finite wavelength, q⃗. This vibration connects two many-body electronic states with different k⃗ vector (verifying ) coupling them through a Peierls effect that is characterized by a quadratic energy surface, as the one shown in Figure 2. Moreover, in the movement from E or M using *Q*_*Rθ*_ (dashed lines), we observe state crossings that are due to the change of electronic state from ferro to antiferrodistortive orbital order.

Looking at the effect of the antiferrodistortive mode on the energy of the various magnetic states, we observe that the stabilization energy from the compressed geometry, Δ_*Rε*_(*c*) (see Table 1), is largest for the FM state, which is consistent with our recent results on layered perovskites like K_2_CuF_4_ or Cs_2_AgF_4_ that also exhibit a spontaneous orthorhombic distortion of MF _6_^4–^ units (M = Cu, Ag) induced by the pseudo JT effect.^15,30^ Globally, the stabilization energy associated with all distortions, Δ_dist_, is quite similar between magnetic states and much larger (≈330 meV) than their separation at any point in the energy surface (≈30 meV) by an order of magnitude. In agreement with Pavarini et al.^16^ this strongly suggests that orbital ordering is controlled by the distortion of the lattice rather than magnetism. However, the AF-A state is stabilized by ≈5 meV with respect to the AF-G one due to the antiferrodistortive *Q*_*Rε*_ mode, although this result is connected to the stronger pseudo JT effect^15^ in the former state rather than to superexchange. Finally, we can observe that the *Q*_*Rθ*_ motion from the E transition state induces a stabilization energy, Δ_*Rθ*_, that is somewhat smaller than Δ_*Rε*_. In this case, the most stable magnetic state is AF-C but, as above, this is connected to the change of distances and the pseudo JT effect.^15^ It is worth noting that layered compounds like K_2_CuF_4_ or Cs_2_AgF_4_ are ferromagnetic^15^ and not antiferromagnetic like KCuF_3_ despite that a local orthorhombic distortion appears in all cases. This obeys the 2D character of the layered compounds which is absent in KCuF_3_ where the interaction between the two closest Cu^2+^ ions placed along the axial (*z*-axis, with a distance of 3.8 Å, see Figure 3) is mainly responsible for this kind of magnetic coupling.

In this work, we have discussed the interaction of ferro- and antiferrodistortive distortions in solids displaying spatial degeneracy. While most models, following Kanamori,^14^ focus on the antiferrodistortive phonon modes, we show here the relevance of the ferrodistortive strain modes. These modes are very important, both quantitatively in the final distortion energy and conceptually, because they are associated with a JT effect in the solid that is completely equivalent to the one in the molecules (warped Mexican hat^1,24^). Moreover, their coupling to antiferrodistortive modes strongly modulates the final geometry and orbital shape of these systems, allowing the prediction of new, low-energy phases that could strongly alter the properties of these complex systems. Initial work^20^ indicates that these results are general and can be applied to other systems including those with nonperovskite structure like rock-salt etc. Also, this coupling has important consequences with regard to orbital-ordering models, where neighboring orbitals are coupled through an effective Heisenberg-like Hamiltonian, which is usually antiferrodistortive. Our calculations show that this first-neighbor coupling in KCuF_3_ (and other lattices with spatial degeneracy are similar^20^) has both ferrodistortive and antiferrodistortive character and that both distortions are cooperative, rather than mutually exclusive, questioning the adequacy of these approaches as they currently stand. Finally, we show that superexchange has very little influence on the state of these systems and that, in fact, orbital ordering models based uniquely on this interaction lead to broken symmetry solutions that are in contradiction with Bloch’s theorem. As a consequence, we believe that they are not adequately founded. We hope that our research helps provide a better understanding of these complex materials.

## Supporting Information Available

Additional computational details, including details of the methods employed, the description of the distortion modes used in Figure 2, and geometries of the main critical points described in that figure (PDF)
