# Supplementary material for: Strain–Phonon Cooperation as a Necessary Ingredient to Understand the Jahn–Teller Effect in Solids
Source: J Phys Chem Lett. 2024 Jun 13;15(25):6476–81. doi: 10.1021/acs.jpclett.4c01256 (PMC11215767; doi:10.1021/acs.jpclett.4c01256)
Supplement: Supplementary file 1 — jz4c01256_si_001.pdf [file jz4c01256_si_001.pdf]

Supporting Information

Strain-Phonon Cooperation as a Necessary Ingredient to Understand the Jahn-Teller Effect in Solids

Toraya Fernández-Ruiz,[1] Inés Sánchez-Movellán,[1] Juan María García-Lastra,[2] Miguel Moreno,[1] José Antonio Aramburu,[1] Pablo García-Fernández,[1] *

[1]*Departamento de Ciencias de la Tierra y Física de la Materia Condensada, Universidad de Cantabria, Cantabria Campus Internacional, Avenida de los Castros s/n, 39005 Santander, Spain*

[2]*Department of Energy Conversion and Storage, Technical University of Denmark, 2800 Kgs. Lyngby, Denmark*

E-mail: garciapa@unican.es

June 12, 2024

1 Computational details

The many-body model of the cooperative Jahn-Teller effect in solids with locally degenerate centers proposed in this letter is supported by first principles methods in the framework of Density Functional Theory (DFT). Assuming periodic boundary conditions, the calculations are carried our using the VASP and CRYSTAL17 codes. The Vienna Ab initio Simulation Package (VASP)[1,2]utilizes a plane wave approach to represent the Bloch orbitals. To explore the interplay of electron and vibrations and their influence on the magnetic characteristics of the system, it becomes imperative to address electron correlation beyond the conventional scope of DFT. Liechtenstein's LDA+U implementation (with U=7.5 eV and J=0.9 eV)[3] and the highly standardized hybrid functional HSE06[4] with a 25% of Hartree-Fock (HF) exchange, both based on the Generalized Gradient Approximation (GGA) approach of Perdew-Burke-Ernzerhof (PBE). The results are also compared with other hybrid functionals as PBE0.[5] This approach enhances the description of these systems as insulators by correcting the intrinsic self-interaction error of DFT. To encompass the full spectrum of geometry distortions and magnetic configurations, we conducted simulations of the cubic Pm-3m and P4/mmm phases of KCuF3 within a tetragonal $\sqrt{2} \times \sqrt{2} \times 2$ supercell, aligning with the configuration employed to characterize the extensively distorted ground state exhibiting I4/mcm symmetry.

The convergence threshold for the electronic self-consistency loop was set at 1×10^{-6} eV, with atomic positions undergoing relaxation via the conjugate gradient algorithm until forces per atom reached below 0.03 eV/Å. Initial orbital occupancies were obtained by a Gaussian smearing with the default value $\sigma = 0.05$. The Brillouin zone of the reciprocal space was sampled with a $2 \times 2 \times 2$ mesh, centered at the Γ point. Even though it may seem not dense enough, it strikes a balance between accuracy and computational cost for the

highly demanding hybrid-functional calculations. Additionally, LDA+U calculations were replicated using a much finer mesh of $8 \times 8 \times 6$, and the results were entirely equivalent. The representation of valence electrons involved utilizing a plane-wave basis set with an energy cutoff of 520 eV, whereas core electrons were characterized using the Projector Augmented Wave method (PAW)[6] in conjunction with pseudopotentials.[7] Specifically, the PAW potentials incorporated 17 and 9 valence electrons for Cu and K cations respectively, 7 for F and Cl, and 6 for O. The 3D representation of the spin density represented in Figure 3 for both stable phases of $KCuF_3$, obtained from VASP, was extracted from the CHGCAR file utilizing the Python tool VASPKIT,[8] and then visualized using the graphical software VESTA.[9]

For comparison purposes, all calculations were executed concurrently utilizing the CRYSTAL17 package.[10] In this software, crystalline orbitals are expanded via a linear combination of Bloch functions, articulated in terms of local functions, which are, in turn, Gaussian type functions. These local functions, available in CRYSTAL website,[11] are defined employing high-quality triple-ζ polarized basis sets, which were developed by Peintinger et al.[12] The hybrid functionals B1WC[13] (including 16% of exact HF exchange), PW1PW[14] (20% HF exchange), HSE06[4] (25% HF exchange) and PBE0[15] (25% HF exchange) were used. To integrate over the first Brillouin zone, an $8 \times 8 \times 8$ grid was utilized. TOLINTEG parameters for real space integrals of the electronic density were set to 9, 9, 9, 9, and 18. The convergence criterion for the energy was established at 10^{-8} Hartree.

2 Definition of distortion modes

The definition of the distortions applied in the $KCuF_3$ lattice are represented in Figure S1. For the tetragonal strain mode $\eta_{\Gamma\theta}$, the distortion is performed by changing the lattice parameters by a quantity η, that allows changing all local octahedra in the cubic perovskite as dictated by the normalized coordinate Q_θ of a local Jahn-Teller tetragonal e_g mode[16] (see Figure S2). That is, this local Q_θ mode in a molecule transforms into the strained $\eta_{\Gamma\theta}$

mode in the Γ point for a solid. The atomic fractional coordinates remain fixed during all the process as corresponds to the application of homogenous strain. On the other hand, for the phonon modes, the lattice parameters remain fixed, while the atomic positions change following the normalized coordinates of a Jahn-Teller tetragonal/orthorhombic e_g mode for the $Q_{R\theta}/Q_{R\varepsilon}$ modes respectively. As it is a phonon in R, both $Q_{R\theta}$ and $Q_{R\varepsilon}$ components of the e_g phonon alternates Q_θ/Q_ε and -Q_θ/-Q_ε local coordinates for neighboring active centers (see Figure S1). This scheme allows characterizing the strain/phonon distortions using a unique criteria (the local JT coordinate) that has been followed to represent Fig. 2 in the main text of the manuscript.

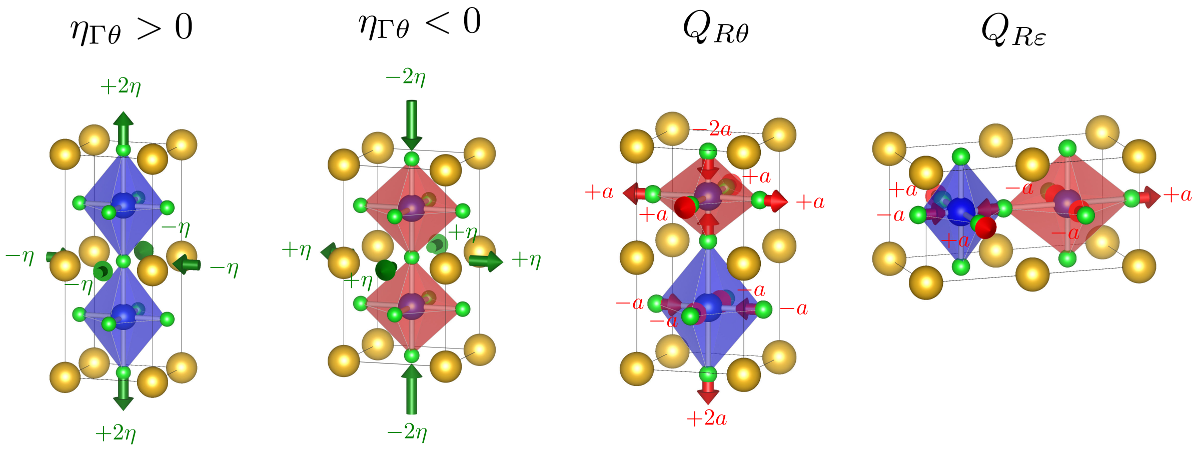

Figure S1: Represetation of the strain (green) and phonon (red) distortions applied in the $KCuF_3$ lattice. Both of them are based on the local distortions of the original Jahn-Teller effect introduced by Bersuker.[16]

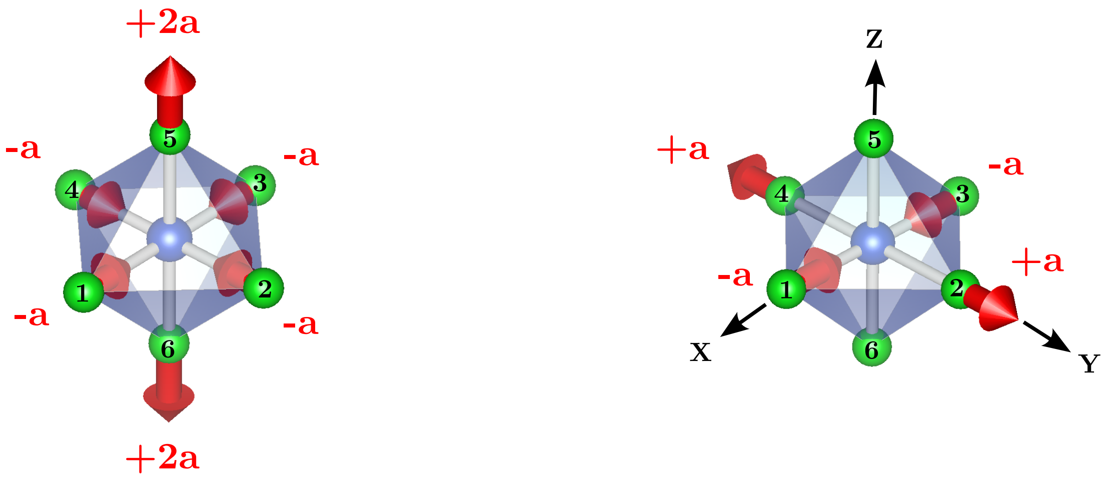

$$Q_\theta = \frac{1}{2\sqrt{3}}(2Z_5 - 2Z_6 - X_1 - Y_2 + X_3 + Y_4) \qquad Q_\varepsilon = \frac{1}{2}(X_1 - Y_2 - X_3 + Y_4)$$

Figure S2: Illustration of the two degenerate components of an e_g mode: Q_θ (tetragonal) and Q_ε (orthorhombic) in an octahedral complex. Quantity a represents the absolute displacement of a single atom. The description of the normalized coordinate of each mode is described at the bottom of the figure. Notation from Bersuker's Jahn-Teller Theory[16]

3 DFT characterization of stationary points

Calculated lattice parameters (a, c) and metal ligand distances (R) for the different phases of $KCuF_3$ are collected in Tables S1 and S2. These results correspond to geometry optimizations of the different phases represented in Figure 2. The energy of each phase is given with respect to the absolute minimum. For both codes, the calculations have been carried out taken symmetry intro account.

Table S1: Optimized geometries of $KCuF_3$ carried out in VASP with HSE06 for the $\eta_{\Gamma\theta} > 0$, $\eta_{\Gamma\theta} < 0$, $\eta_{\Gamma\theta} > 0 + Q_{R\theta}$ and $\eta_{\Gamma\theta} < 0 + Q_{R\varepsilon}$ geometries. The energy is given with respect to the absolute $\eta_{\Gamma\theta} < 0 + Q_{R\varepsilon}$(AF-A) minimum. All the energies are given per formula unit.

Elongated tetragonal strain ($\eta_{\Gamma\theta} > 0$)					
Magnetic order	a(Å)	c(Å)	R_{EQ} (Å)	R_{AX} (Å)	ΔE (meV)
FM	5.5930	8.7321	1.977	2.183	225.1
AF-A	5.6073	8.6815	1.982	2.170	224.8
AF-C	5.5798	8.7268	1.972	2.181	177.7
AF-G	5.5796	8.7258	1.972	2.181	177.1

Compressed tetragonal strain ($\eta_{\Gamma\theta} < 0$)					
Magnetic order	a(Å)	c(Å)	R_{EQ} (Å)	R_{AX} (Å)	ΔE (meV)
FM	5.9030	7.7837	2.087	1.946	253.5
AF-A	5.9037	7.7756	2.087	1.944	211.9
AF-C	5.9053	7.7793	2.087	1.945	249.0
AF-G	5.9008	7.7792	2.086	1.945	209.5

Elongated tetragonal strain+AFD tetragonal phonon ($\eta_{\Gamma\theta} > 0 + Q_{R\theta}$)							
Magnetic order	a(Å)	c(Å)	$R_{EQ,c}$(Å)	$R_{AX,c}$(Å)	$R_{EQ,e}$(Å)	$R_{AX,e}$(Å)	ΔE (meV)
FM	5.7126	8.323	2.116	1.8707	1.923	2.291	46.2
AF-A	5.7129	8.324	2.116	1.8702	1.923	2.292	47.6
AF-C	5.7119	8.322	2.118	1.8665	1.920	2.295	25.5
AF-G	5.7118	8.327	2.118	1.8663	1.920	2.297	28.3

Compressed tetragonal strain+AFD orthorhombic phonon ($\eta_{\Gamma\theta} < 0 + Q_{R\varepsilon}$)						
Magnetic order	a(Å)	c(Å)	R_L (Å)	R_S (Å)	R_{AX} (Å)	ΔE (meV)
FM	5.9279	7.9339	2.285	1.906	1.983	29.3
AF-A	5.9424	7.9037	2.297	1.904	1.975	0
AF-C	5.9282	7.9341	2.286	1.906	1.983	28.7
AF-G	5.9321	7.9153	2.289	1.905	1.978	4.4

Table S2: Lattice parameters and metal-ligand distances for $KCuF_3$ optimized by CRYSTAL17 using HSE06 hybrid functional for the $\eta_{\Gamma\theta} > 0$, $\eta_{\Gamma\theta} < 0$, $\eta_{\Gamma\theta} > 0 + Q_{R\theta}$ and $\eta_{\Gamma\theta} < 0 + Q_{R\varepsilon}$ phases. The energy is given with respect to the absolute $\eta_{\Gamma\theta} < 0 + Q_{R\varepsilon}$(AF-A) minimum. All the energies are given per formula unit.

Elongated tetragonal strain ($\eta_{\Gamma\theta} > 0$)					
Magnetic order	a(Å)	c(Å)	R_{EQ} (Å)	R_{AX} (Å)	ΔE (meV)
FM	5.542	8.616	1.9594	2.1539	205.9
AF-A	5.542	8.615	1.9595	2.1539	205.9
AF-C	5.526	8.648	1.9536	2.1622	159.0
AF-G	5.526	8.648	1.9536	2.1621	158.6

Compressed tetragonal strain ($\eta_{\Gamma\theta} < 0$)					
Magnetic order	a(Å)	c(Å)	R_{EQ} (Å)	R_{AX} (Å)	ΔE (meV)
*FM	-	-	-	-	-
AF-A	5.856	7.679	2.0706	1.9196	169.0
AF-C	5.856	7.695	2.0702	1.9239	204.2
AF-G	5.857	7.676	2.0708	1.9189	166.8

*FM becomes cubic after geometry optimization

Elongated tetragonal strain+AFD tetragonal phonon ($\eta_{\Gamma\theta} > 0 + Q_{R\theta}$)							
Magnetic order	a(Å)	c(Å)	$R_{EQ,c}$(Å)	$R_{AX,c}$(Å)	$R_{EQ,e}$(Å)	$R_{AX,e}$(Å)	ΔE (meV)
FM*	5.5154	8.5200	2.214	1.962	2.046	2.298	45.8
AF-A*	5.5154	8.5200	2.214	1.962	2.046	2.298	47.5
AF-C	5.7057	8.2591	2.236	1.931	1.9275	2.242	21.8
AF-G*	5.5154	8.5200	2.214	1.962	2.046	2.298	25.3

*FM, AF-A, AF-G: geometry corresponds to the minimum of single-point calculations

Compressed tetragonal strain+AFD orthorhombic phonon ($\eta_{\Gamma\theta} < 0 + Q_{R\varepsilon}$)						
Magnetic order	a(Å)	c(Å)	R_L (Å)	R_S (Å)	R_{AX} (Å)	ΔE (meV)
FM	5.8224	7.9190	2.227	1.890	1.980	25.1
AF-A	5.8410	7.8703	2.240	1.890	1.968	0
*AF-C	-	-	-	-	-	-
AF-G	5.8377	7.8378	2.240	1.888	1.971	4.5

*AF-C: geometry optimization for AF-C order in $\eta_{\Gamma\theta} < 0 + Q_{R\varepsilon}$ phase spontaneously evolves to $\eta_{\Gamma\theta} > 0 + Q_{R\theta}$ phase. This is not surprising as, for this magnetic order, the energy of $\eta_{\Gamma\theta} > 0 + Q_{R\theta}$ phase is lower than that of $\eta_{\Gamma\theta} < 0 + Q_{R\varepsilon}$ by 3.2 meV as derived from VASP calculations (see Table S1)

4 Acknowledgements

We acknowledge financial support from Grant No. PID2022-139776NB-C63 funded by MCIN/AEI/10.13039/501100011033. T. F.-R. (grant PRE2019-089054) acknowledges financial support from Ministerio de Ciencia, Innovación y Universidades while I. S.-M. (grant BDNS:589170) acknowledges financial support from Universidad de Cantabria and Gobierno de Cantabria. T. Fernández-Ruiz and I. Sanchez-Movellán contributed equally to this work.

References

(1) Kresse, G.; Hafner, J. Ab initio molecular-dynamics simulation of the liquid-metal–amorphous-semiconductor transition in germanium. *Phys. Rev. B* **1994**, *49*, 14251–14269.

(2) Kresse, G.; Furthmüller, J. Efficient iterative schemes for ab initio total-energy calculations using a plane-wave basis set. *Phys. Rev. B* **1996**, *54*, 11169–11186.

(3) Liechtenstein, A.; Anisimov, V.; Zaanen, J. *Phys. Rev. B* **1995**, *52*, 5467–5470.

(4) Heyd, J.; Scuseria, G. E.; Ernzerhof, M. Hybrid functionals based on a screened Coulomb potential. *J. Chem. Phys.* **2003**, *118*, 8207–8215.

(5) Adamo, C.; Barone, V. Toward reliable density functional methods without adjustable parameters: The PBE0 model. *J. Chem. Phys.* **1999**, *110*, 6158–6170.

(6) Blöchl, P. E. Projector augmented-wave method. *Phys. Rev. B* **1994**, *50*, 17953.

(7) Kresse, G.; Joubert, D. From ultrasoft pseudopotentials to the projector augmented-wave method. *Phys. Rev. B* **1999**, *59*, 1758.

(8) Wang, V.; Xu, N.; Liu, J.-C.; Tang, G.; Geng, W.-T. VASPKIT: A user-friendly interface facilitating high-throughput computing and analysis using VASP code. *Comp. Phys. Comm.* **2021**, *267*, 108033.

(9) Momma, K.; Izumi, F. VESTA: a three-dimensional visualization system for electronic and structural analysis. *J. App. Cryst.* **2008**, *41*, 653–658.

(10) Dovesi, R.; Erba, A.; Orlando, R.; Zicovich-Wilson, C. M.; Civalleri, B.; Maschio, L.; Rérat, M.; Casassa, S.; Baima, J.; Salustro, S. et al. Quantum-mechanical condensed matter simulations with CRYSTAL. *WIREs: Comp. Mol. Sci.* **2018**, *8*, e1360.

(11) CRYSTAL Basis Sets. `https://www.crystal.unito.it/basis_sets.html`, 2023; Accessed: January 2023.

(12) Peintinger, M. F.; Oliveira, D. V.; Bredow, T. Consistent Gaussian basis sets of triple-zeta valence with polarization quality for solid-state calculations. *J. Comp. Chem.* **2013**, *34*, 451–459.

(13) Bilc, D. I.; Orlando, R.; Shaltaf, R.; Rignanese, G. M.; Iniguez, J.; Ghosez, P. Hybrid exchange-correlation functional for accurate prediction of the electronic and structural properties of ferroelectric oxides. *Phys. Rev. B* **2008**, *77*.

(14) Bredow, T.; Gerson, A. R. Effect of exchange and correlation on bulk properties of MgO, NiO, and CoO. *Phys. Rev. B* **2000**, *61*, 5194–5201.

(15) Adamo, C.; Barone, V. Toward reliable density functional methods without adjustable parameters: the PBE0 model. *J. Chem. Phys* **1999**, *110*.

(16) Bersuker, I. B. *The Jahn-Teller Effect*; Cambridge University Press: Cambridge, 2006.
